# Organizational aspects of intensive care unit resource allocation in a primary respiratory intensive care center specialized for the treatment of SARS-COV-2 patients

**DOI:** 10.3325/cmj.2020.61.304

**Published:** 2020-06

**Authors:** Jasminka Peršec, Andrej Šribar, Tatjana Kereš

**Affiliations:** 1 Clinical Department of Anesthesiology, Resuscitation and Intensive Care Medicine, University Hospital Dubrava, Zagreb, Croatia; 2 School of Dental Medicine, University of Zagreb, Zagreb, Croatia *andrej.sribar@gmail.com*; 3 Clinical Department of Internal Medicine, University Hospital Dubrava, Zagreb, Croatia

In the early phase of the severe acute respiratory syndrome coronavirus 2 (SARS-COV-2) pandemic in Croatia, in March 2020, the Croatian Ministry of Healthcare designated University Hospital Dubrava (*Klinička bolnica Dubrava*, KBD) as the primary respiratory intensive center for the treatment of SARS-COV-2 patients from seven administrative regions of central and northwestern Croatia. Two subunits have been formed: respiratory center (RC) for treatment of patients with a moderate clinical course who require conservative treatment coupled with supplemental oxygen; and respiratory intensive care center (RIC) specialized in mechanical ventilation and other critical care procedures (such as vasoactive drug administration, continuous renal replacement therapy, blood purification, and extracorporeal membrane oxygenation).

## Infection containment measures and decontamination protocol

KBD is comprised of three units – unit A (psychiatric clinic), unit B (one-day surgery and extracorporeal shock-wave lithotripsy), and unit C (main building). The units A and B were repurposed for the initial treatment of COVID-19 patients, while the Croatian Army set up tents for patients with a moderate disease course. In order to avoid the viral spread to the unit C, especially with known data about surface survival of SARS-COV-2 (1), special entry and exit protocols were established.

Unidirectional negative pressure airlock chamber was placed at the entry point in the basement corridor between the unit C (clean unit) and units A and B (contaminated units). Before airlock entry, the personnel remove all belongings (such as jewelry, watches, and mobile phones) and put on a single-use personal protective equipment (PPE) set, which is comprised of protective pants, boot covers, gown, hood, FFP3 mask (leak test is performed after mask fitting), eye protection goggles, and a double pair of nitrile gloves (one pair is worn under the gown cuff, and the other over the gown cuff).

After the transition through the airlock, healthcare personnel enter the contaminated area, where they participate in diagnostic and therapeutic procedures with COVID-19 patients. During this period no activities that may cause contamination are allowed (such as touching the face or removing PPE). After the end of the shift, the personnel exit the building, remove the outer pair of gloves, while the inner pair is disinfected and left for 10 minutes in the open air to minimize aerosol generation. Subsequently, they gradually remove PPE according to predefined protocols and take a hot water and octenidine dihydrochloride gel shower lasting at least 20 minutes.

## Organization of intensive care unit procedures and diagnostic tests in the respiratory intensive care center

The most important clinical complication of COVID-19 requiring intensive requiring care unit (ICU) admission is bilateral viral pneumonia, which causes acute respiratory distress syndrome (ARDS) with hypoxemic respiratory failure. The population at risk are elderly patients with multiple comorbidities (2,3). In addition to pharmacotherapy, a primary supportive measure in these patients is mechanical ventilation. The preferred airway management method of choice is endotracheal intubation with all the aerosol-related prerequisites (adequate sedation and neuromuscular blockade, closed suction systems, and additional PPE), although the consensus still has not been achieved on the safety of high-flow nasal oxygen and non-invasive ventilation. The level of positive end expiratory pressure (PEEP) is crucial in ventilatory management of these patients in order to maintain adequate lung aeration and avoid ventilator-induced lung injury. The “optimal” level of PEEP may be determined by various methods, with electrical impedance tomography (EIT) and esophageal manometry (EM) being the gold standards in ICU practice. However, due to increased nursing workload and exposure (EIT) and increased probability of aerosol generation (EM), *ARDSNet* PEEP/FIO_2_ tables (4), ie, expert consensus on the choice of the minimum PEEP and accompanying fraction of inspired oxygen needed to achieve oxygen saturation of 88-92%, are used in order to minimize the risk of contamination, with an acceptable success rate.

In order to improve gas exchange and decrease shunting in patients with severe ARDS, prone 12-hour sessions of position ventilation are performed, and positioning is performed during nurse shift change (5). In the minority of patients in whom mechanical ventilation is not adequate *per se*, veno-venous extracorporeal membrane oxygenation may be initiated (although there are conflicting data in these patients) (6), with surgeons and perfusionists available on call.

In order to minimize the exposure of other healthcare personnel (such as laboratory and radiologic technicians), point-of-care diagnostic tests are used whenever appropriate. Ultrasound is the preferred method of choice for the assessment of the lung (eliminating the need for chest X-ray) and cardiac function, as well as volume status, and is routinely used for arterial and venous access to minimize the number of cannulation attempts and time spent in direct contact with the patient.

If there is a need for non-bedside laboratory diagnostic tests, special containers are placed in the open air, outside of the contaminated area, where samples are stored in a protocolized manner, and are collected by laboratory personnel with PPE ten minutes after the aerosol has settled.

All the described protective measures have resulted in zero COVID-19 infections of KBD RIC personnel as of May 31, 2020, and we hope that strict adherence to these protocols will help keep this number low.
